# Co-contraction characteristics of lumbar muscles in patients with lumbar disc herniation during different types of movement

**DOI:** 10.1186/s12938-018-0443-2

**Published:** 2018-01-24

**Authors:** Wenjing Du, Huihui Li, Olatunji Mumini Omisore, Lei Wang, Wenmin Chen, Xiangjun Sun

**Affiliations:** 1 Institute of Biomedical and Health Engineering, Shenzhen Institutes of Advanced Technology, Chinese Academy of Sciences, 1068 Xueyuan Boulevard, University Town of Shenzhen, Xili Nanshan, Shenzhen, 518055 China; 2 Shenzhen College of Advanced Technology, University of Chinese Academy of Sciences, Shenzhen, 518055 China; 3 Jiangxi University of Science and Technology, Jiangxi, China

**Keywords:** Lumbar spine, Co-contraction ratio, Lumbar disc herniation, Lumbar muscle activity

## Abstract

**Background:**

Muscular performance is an important factor for the mechanical stability of lumbar spine in humans, in which, the co-contraction of lumbar muscles plays a key role. We hypothesized that when executing different daily living motions, the performance of the lumbar muscle co-contraction stabilization mechanism varies between patients with lumbar disc herniation (LDH) and healthy controls. Hence, in this study, co-contraction performance of lumbar muscles between patients with LDH and healthy subjects was explored to check if there are significant differences between the two groups when performing four representative movements.

**Methods:**

Twenty-six LDH patients (15 females, 11 males) and a control group of twenty-eight subjects (16 females, 12 males) were recruited. Surface electromyography (EMG) signals were recorded from the external oblique, lumbar multifidus, and internal oblique/transversus abdominis muscles during the execution of four types of movement, namely: forward bending, backward bending, left lateral flexion and right lateral flexion. The acquired EMG signals were segmented, and wavelet decomposition was performed followed by reconstruction of the low-frequency components of the signal. Then, the reconstructed signals were used for further analysis. Co-contraction ratio was employed to assess muscle coordination and compare it between the LDH patients and healthy controls. The corresponding signals of the subjects in the two groups were compared to evaluate the differences in agonistic and antagonistic muscle performance during the different motions. Also, sample entropy was applied to evaluate complexity changes in lumbar muscle recruitment during the movements.

**Results:**

Significant differences between the LDH and control groups were found in the studied situations (p < 0.05). During the four movements considered in this study, the participants of the LDH group exhibited a higher level of co-contraction ratio, lower agonistic, and higher antagonistic lumbar muscle activity (p < 0.01) than those of the control group. Furthermore, the co-contraction ratio of LDH patients was dominated by the antagonistic muscle activity during the movements, except for the forward bending motion. However, in the healthy control group, the agonistic muscle activity contributed more to the co-contraction ratio with an exception for the backward bending motion. Conversely, the sample entropy value was significantly lower for agonistic muscles of LDH group compared to the control group (p < 0.01) while the entropy value was significantly greater in antagonistic muscles (p < 0.01) during the four types of movement, respectively.

**Conclusions:**

Lumbar disc herniation patients exhibited numerous variations in the evaluated parameters that reflect the co-contraction of lumbar muscles, the agonistic and antagonistic muscle activities, and their respective sample entropy values when compared with the healthy control group. These variations could be due to the compensation mechanism that was required to stabilize the spine. The results of this study could facilitate the design of efficient rehabilitation methods for treatment of lumbar muscle dysfunctions.

## Background

Muscle co-contraction, the simultaneous activation of antagonistic and agonistic muscles, plays an important role in stiffening and stabilizing lumbar spine to minimize the effect of potential internal and external disturbances on the posture, maintaining moments, or regulating the loads at the spine [1–3]. According to Panjabi et al. [4], spinal stability is ensured by the simultaneous contribution of passive, active, and neural control subsystems. Among them, the active subsystem, which is formed by the muscles around the spine, plays more than 80% of the role in maintaining lumbar spine stability [5, 6]. These muscles have been categorized as global and local ones [7]. Some of them maintain long-lasting contractions which are essential for the human upright position [8]. The global muscles, including the external oblique and the rectus abdominal muscles, span multiple spinal segments which enables them to produce torque and ensure general trunk stabilization. On the other side, the local muscles such as lumbar and paraspinal muscles [9–11] control the spine curvature, ensure sagittal and lateral stiffness to maintain the mechanical stability of the lumbar spine [8]. Lumbar disc herniation (LDH) is a medical condition in which a tear in the fibrous ring around the lower lumbar vertebral disc allows the soft central portion to bulge out. Recently, it is becoming a widespread medical problem with most people affected being around the ages of 20–50 [12, 13]. LDH can result from general wear and tear during daily activities, such as constant sitting, running, and driving, or a sedentary lifestyle or in case of extreme actions such as lifting of heavy loads. Low back pain and radiculopathy are common symptoms among the patients suffering from LDH.

Stability describes the ability to maintain equilibrium despite the presence of kinematic and/or control disturbances. The mechanical stability of lumbar spine is considered an important factor [14] for ensuring the balance and posture required for normal daily activities such as walking, running, flexion, and extension [15]. Particularly, lumbar spine instability determines an increased risk of recurrent lumbar spine disc herniation, which, subsequently, often leads to disorders in the lower limbs loading, impairs proprioception and deteriorates postural stability [16]. The presence of pain also affects maintaining the mechanical stability of the lumbar spine. Thus, analyzing the characteristics of co-contraction of lumbar muscles in different subject groups can be helpful in understanding the control mechanisms of their neuromuscular systems. The preconditions to ensuring lumbar spine stability could be taken into account when performing exercises to improve spinal stability and when exploring LDH disorders [17].

There are numerous studies on the co-contraction of the neck, and lower limb muscles [18–20], and researchers have indicated that the regulation of the co-contraction is presumably an efficient adjusting mechanism for ensuring the stability of neck and joints. For the coordination of lumbar muscles, several researchers have proved that the co-contraction can be affected by movement speed [18], posture [21] and task load [22]. Surface electromyography (sEMG) is commonly used to quantify the co-contraction of muscles [23, 24]. The biomechanical analysis reveals that neglecting co-contraction performance during dynamic motor activities will result in spinal load underestimation [25]. Co-contractions during maximal trunk movements (flexion, lateral bending, and axial twisting) as well as combinations of lumbar and thoracic movements were investigated by Silvestri et al. [26]. In most cases, studies related to the co-contraction of trunk muscles were conducted on healthy subjects under isometric flexion and extension [25], or on subjects suffering from low back pain during sitting [27]. However, the lumbar muscle co-contraction performance while executing representative motions of daily living is yet to be fully explored.

Hence, the aim of this study is to investigate whether the co-contraction performance of lumbar muscles differs between LDH subjects and healthy controls. Effects of antagonistic and agonistic muscle activities and recruitment patterns on the co-contraction of lumbar muscles are also investigated. All findings reported in this study are based on analysis of sEMG signals acquired during the execution of four specific types of movement.

## Methods

### Participants

In this study, a total of 54 subjects from department of local rehabilitation, Longgang Center Hospital (Shenzhen, China) were recruited. Twenty-six of the subjects were LDH patients who suffered from low back pain in the last 3–12 months before collecting their sEMG data, and their LDH was diagnosed by means of computed tomography (CT) or magnetic resonance imaging (MRI). Furthermore, the subjects had no history of spinal surgery, lumbar spine or hip contractures, chronic pain pathology, respiratory, neurological or cardiac diseases, and usually used physical therapy to relieve the pain symptoms. The remaining 28 subjects were healthy controls who had no history of LDH. The subjects in the control group were selected such that their gender, age, weight, height, and body mass index (BMI) matched the ones of the subjects of the LDH group. Details about subjects are presented in Table 1.

**Table 1:** Details about subjects of both groups (means [SD])

	Female (57.41%)			Male (42.59%)		
	Control (29.63%) (n = 16)	LDH (27.78%) (n = 15)	*p* value	Control (22.22%) (n = 12)	LDH (20.37%) (n = 11)	*p* value
Age (years)	36.06 (4.53)	39.40 (6.43)	0.104	34.50 (6.97)	39.91 (10.79)	0.165
Age range (years)	30–47	28–50	–	28–50	22–50	–
Body weight (kg)	56.94 (10.17)	55.20 (5.43)	0.561	64.83 (6.45)	69.27 (4.52)	0.072
Body height (cm)	159.94 (4.92)	158.87 (4.12)	0.518	172.75 (5.56)	176.27 (4.08)	0.100
BMI (kg/m^2^)	22.32 (4.43)	21.87 (1.98)	0.720	21.72 (1.89)	22.29 (1.27)	0.410
BMI range (kg/m^2^)	16.45–30.11	18.43–25.15	–	19.27–24.77	20.34–23.84	–
Pain VAS	–	32.00 (15.09)	–	–	42.73 (18.49)	–

*SD* standard deviation, *BMI* body mass index, *VAS* visual analogue scale

The experimental procedure was approved by the Institutional Review Board of Shenzhen Institutes of Advanced Technology (Reference No. SIAT-IRB-140215-H0037), and all the subjects signed an informed consent forms prior to testing. One-way analysis of variance (ANOVA) was used to determine whether there are any statistically significant differences (significant level p < 0.05) between the mean of age or BMI in both groups. The results are shown in Table 1. Also, the pain intensity of subjects in the LDH group was evaluated using a 100-mm Visual Analogue Scale (VAS) with 0 indicating no pain and 100 indicating an unbearable (but imaginable) pain.

### Experimental design and sEMG signal collection

**Fig. 1:**
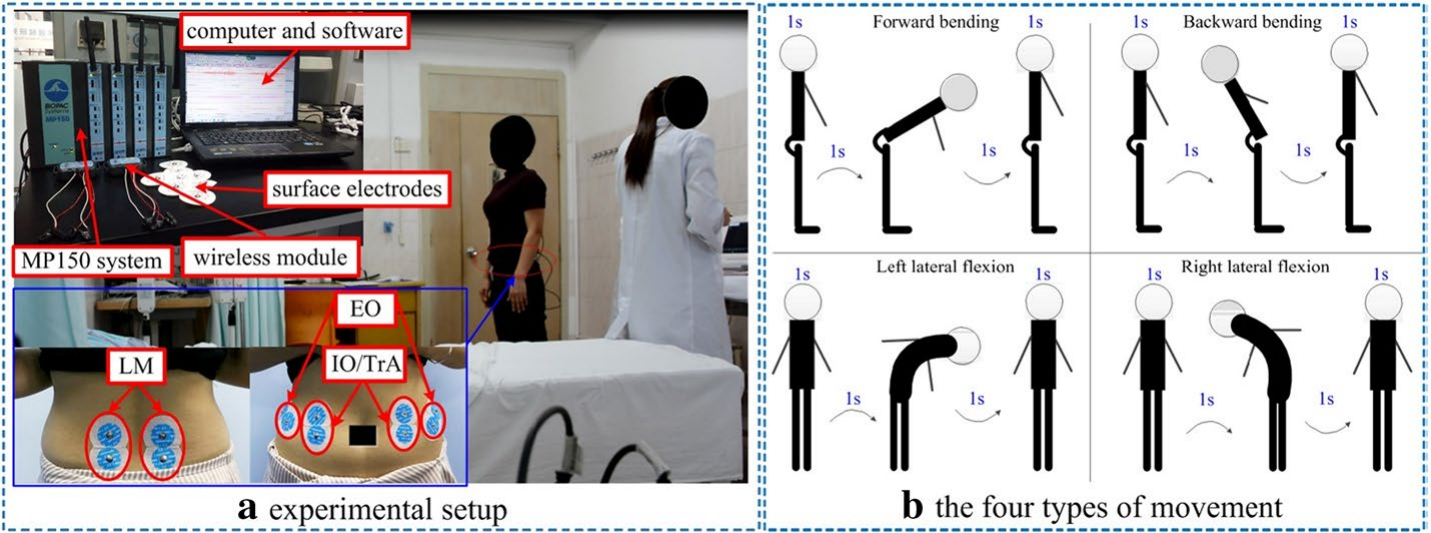
**a** Experimental setup, **b** the four types of movement

All the participants were asked to perform four types of movement namely: forward bending, backward bending, left lateral flexion and right lateral flexion. Each of the movements was performed three times in a sequence and each time, a separate sEMG recording was captured. Thus, 12 sEMG recordings in total were captured for each subject. All subjects performed the required actions under the guidance of an examiner and an automated system that produced rhythmic audio signals to ensure consistency in movement pace. The experimental setup is illustrated in Fig. 1a. Each subject was asked to stand straight on a horizontal ground for 1 s with hands kept down. Sequel to this, the subject listened to the audio rhythm to perform the required movements. Each movement consisted of four sub-parts, namely: standing upright, bending forward, going back to standing position, and standing upright again. Each sub-part took about 1 s to execute. Thus, each subject performed the corresponding motions for approximately 4 s, after which he/she was given rest for another 30 s before proceeding to the next movement. The movements and their subparts are illustrated in Fig. 1b.

According to clinical observations, subjects with LDH have weaker lumbar muscles than healthy subjects, and as a result, they cannot perform large-degree flexion. Hence, subjects in the LDH group were asked to try their best in completing the left and right lateral flexion movements unless feeling any pain. Six pairs of surface electrodes (disposable Ag/AgCl, 10 mm diameter, LT-301, China), were placed on the subject’s waist covering the muscles of interest: left and right external oblique (EO), lumbar multifidus (LM), and internal oblique/transversus abdominis (IO/TrA) muscles. As shown in Fig. 1a, only one electrode pair was required to cover each pair IO/TrA muscles since the muscles pass through a common region around the human waist. Before each session of the experiment, the subject’s skin at the sites of electrode attachment was cleaned with alcohol to ensure that the electrode–skin contact cannot be affected by contamination. The center-to-center distance between the electrodes of each channel was around 20 mm. Finally, data were acquired at a sampling rate of 1000 Hz using a configurable electromyography (EMG) system (BioNomadix, BIOPAC Systems, Inc., Goleta, CA, USA).

### Signal processing

**Fig. 2:**
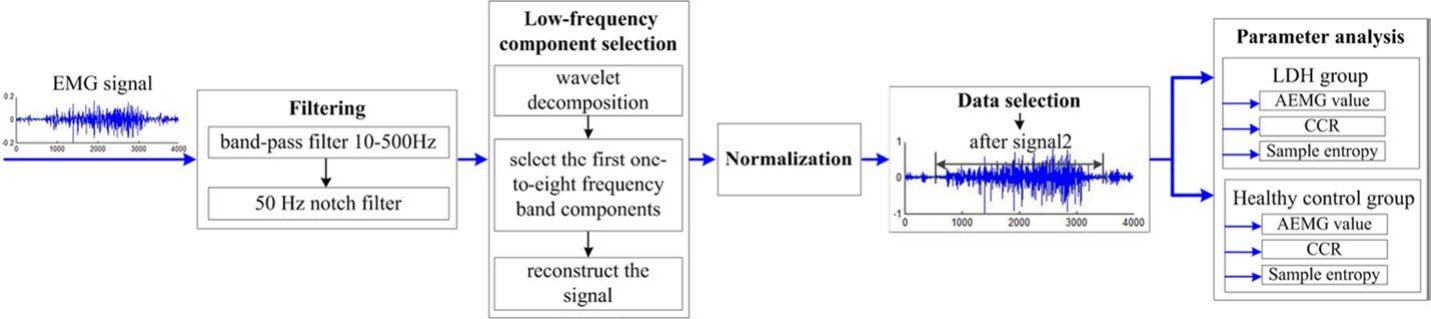
sEMG signal pre-processing

Before analyzing the collected data, pre-processing was performed as illustrated in Fig. 2.

#### Filtering

The typical band of sEMG signals is in the range 10–500 Hz. Therefore, first, a band-pas filter in the said band was applied over the acquired sEMG signals. Afterwards, a 50 Hz-notch filter was used to eliminate the power–frequency disturbances. Both filters were applied offline using MATLAB 2013a. Since the most of the energy of the sEMG signal is focused in its low-frequency components, in the range of 20–150 Hz [28, 29], to determine and isolate those components in the particular case, the signals were decomposed by means of wavelet packet decomposition, and the appropriate low-frequency components were then used to reconstruct the signal.

#### Wavelet packet analysis

The decomposition procedure for the sEMG signal can be described as follows:

Given as sEMG signal S, it is decomposed by wavelet packet method into a sum of $2^{m}$ signal components:

1 $${\text{S}} = {\text{S}}_{1} + {\text{S}}_{2} + \cdots \cdots \cdots + {\text{S}}_{r}$$

where ${\text{r}} = 1, 2, \ldots , 2^{m} ,$ and m is the decomposition level that can take values in the range ${\text{n}} = 1, 2, \ldots , {\text{i }}$, and n represents the number of samples within a frequency band. Therefore, the frequency components of the signal that corresponds to each movement $(s_{r,n} )$ are given as:

2 $${\text{S}}_{r,n} = \left[ {{\text{S}}_{r,1} ,{\text{S}}_{r,2} , \ldots ,{\text{S}}_{r,n} , \ldots ,{\text{S}}_{r,i} } \right]$$

To verify the energy distribution in the sEMG signal, after the decomposition, the wavelet packet energy P can be expressed as a superposition of the signal components:

3 $${\text{P}} = \mathop \sum \limits_{r = 1}^{{2^{m} }} E_{r} = \mathop \sum \limits_{r = 1}^{{2^{m} }} \left| {\mathop \sum \limits_{n = 1}^{i} \left| {s_{r,n} } \right|^{2} } \right|^{2}$$

Furthermore, wavelet packet energy spectrum (WPES) is employed to describe the energy distribution of the sEMG signal on each frequency band. The WPES on each frequency band can be defined as:

4 $$ps_{r} = E_{r}$$

Finally, the WPES can be presented as a one-dimensional array:

5 $${\text{WPES}} = \left\{ {ps_{1} ,ps_{2} , \ldots \ldots \ldots .,ps_{{2^{m} }} } \right\}$$

The acquired and filtered sEMG signals were decomposed using four-level decomposition [30] as described above. Having the highest signal frequency being 500 Hz and a total number of frequency bands of with $2^{m} = 16$, the lowest frequency range was 0–31.25 Hz. We found that for all participants, the components from one to eight held more than 80% of the signal energy, as illustrated in Table 2. We then used these components for reconstruction of the sEMG signal.

**Table 2:** Total WPES of components from one to eight

	LDH						Healthy controls					
	Left side (%)			Right side (%)			Left side (%)			Right side (%)		
	EO	IO/TrA	LM	EO	IO/TrA	LM	EO	IO/TrA	LM	EO	IO/TrA	LM
Forward bending	89.32	82.10	87.64	88.33	86.33	88.70	90.66	90.02	86.67	89.65	86.70	85.79
Backward bending	91.90	88.93	81.64	90.00	89.30	83.81	94.76	86.32	86.91	91.04	87.98	85.15
Left lateral flexion	84.73	85.11	85.03	87.74	86.70	83.60	90.23	87.93	89.10	90.45	85.19	84.99
Right lateral flexion	89.43	87.58	86.61	83.95	80.64	87.56	91.67	84.09	83.69	86.98	88.75	87.09

The electromyographic acquisition from upper trunk muscles is often accompanied by low-frequency electrocardiographic (ECG) noise, due to the proximity of electrodes to the heart [31]. Also, most of the ECG signal energy is located in the range between 0.5 and 35 Hz [32, 33]. Hence, ECG noise in the acquired data was attenuated using a 35–250 Hz band-pass filter.

Finally, the sEMG data were exported as a MATLAB file for the further processing.

#### SEMG signal normalization

**Fig. 3:**
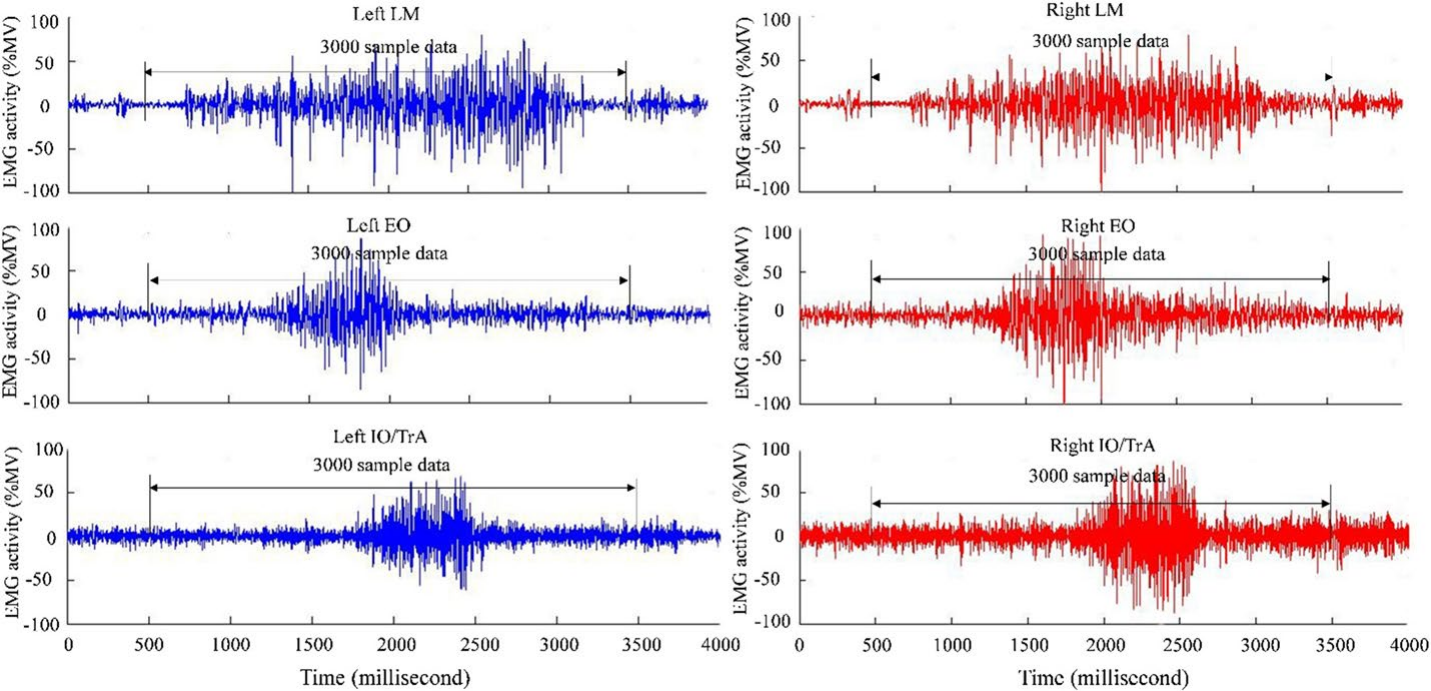
sEMG activity of six muscles from left and right lumbar of one subject while forward bending movement

According to existing literature, maximum voluntary contraction (MVC) is a commonly used method for sEMG normalization [34]. The method involves rating the energy level of each subject based on the maximum contraction possible for a reference muscle. However, it might not be suitable for LDH subjects as the lumbar vertebra can be seriously hunted when trying to evaluate the maximum possible contraction. An alternative approach, which was adopted for sEMG normalization in this study, is the maximum value (MV) method [35]. Simply, this post-processing method involves determining the peak value of a sEMG signal for a distinct movement recorded from a subject. Then, this value can be used to normalize rest of the data series such that these parts are expressed as a percentage of the MV (%MV). This normalization procedure was used to ensure that a common ground is established when comparing signal from all subjects irrespective of their LDH status. Sequel to acquisition of sEMG signals, respective data for each movement made by each subject were normalized based on the individual MV. Each data sample had a length of 4000 ms; however, data with length of 3000 ms, selected per each sample, were applied for subsequent processing. For example, the signal selected from one subject from all the six channels captured during forward bending movement is as shown in Fig. 3. sEMG activity of the involved muscles was standardized so that the sEMG-amplitude relation could be compared between LDH and healthy control subjects.

### Parameters

#### Average electromyogram values and co-contraction ratio

For each type of movement made by each subject, an average electromyogram (AEMG) was calculated as the mean of amplitudes of the sEMG signal from all the three trials. Hence, AEMG is a single-valued parameter that is not associated with time series of the sEMG signal. However, it is a very important time-domain index in the selected length of the sEMG signal because it reflects innervation input from sEMG signal in all explored muscles during the trunk actions. The derived AEMG value can be expressed as:

6 $${\text{AEMG}} = \frac{{\mathop \sum \nolimits_{i = 1}^{N} \left| {Data[i]} \right|}}{N}$$

Muscle co-contraction is an important variable which can be used to assess lumbar muscle function. Of the existing co-contraction methods that are commonly used to quantify co-contraction of muscle activities, the ratio of antagonistic activity to total muscle activities [36], and two times the activities of the antagonistic muscles normalized to sum of the activities of the muscles [37] are commonly used methods. Also, the proportion of co-contraction muscle forces to total muscle forces was used by Choi [38] to quantify the co-contraction of muscle activities. In this study, co-contraction ratio (CCR) was calculated as normalized integration of the antagonistic sEMG activities divided by that of the total muscle activities, as given in Eq. 7, where $AEMG_{A}$ is the normalized integration of antagonistic muscle activities, and $AEMG_{B}$ is the normalized integration of agonistic muscle activities.

7 $${\text{CCR}} = \frac{{AEMG_{A} }}{{AEMG_{ B} + AEMG_{A} }}$$

As shown in Fig. 1, the six muscles considered in this study were attributed to either agonistic or antagonistic muscles. The index ‘agonistic’ simply refers to muscles at the side of the body where they are assumed to support axial torque, and the index ‘antagonistic’ refers to muscles at the opposite side, and produce an opposing joint torque to the agonist muscles. For instance, during the forward bending movement, the agonistic muscles are the left external oblique (L_EO), right external oblique (R_EO), left internal oblique/transversus abdominis (L_IO/TrA) and right internal oblique/transversus abdominis (R_IO/TrA), while the antagonistic muscles are the left and right lumbar multifidus (L_LM and R_LM). However, for backward bending movement, the agonists are the L_LM and R_LM [39], while the antagonists are L_EO, L_IO/TrA, R_EO and R_IO/TrA. Similarly, the agonists for left lateral flexion are L_LM, L_EO and L_IO/TrA while R_LM, R_EO and R_IO/TrA are the antagonists. And lastly, for the right lateral flexion, these agonistic muscles are R_LM, R_EO and R_IO/TrA [40], while the antagonistic muscles are L_LM, L_EO and L_IO/TrA.

#### Sample entropy

Sample entropy is the negative natural logarithm that shows the conditional probability that a subseries of length m that matches point-wise within a tolerance r will also match at the next segment within the series [41]. As a method for estimating the complexity and regularity of biomedical signal in a certain series, sample entropy shows greater advantage over its counterpart, approximate entropy, as it is independent of the time series, avoids bias by counting self-matches, and requires lower execution time. When a muscle activity occurs, the complexity of the system is usually accompanied by noticeable change in the sEMG signal. In this study, we explored the change in complexity of lumbar muscle activities by using the sample entropy of the sEMG signal during four movements. The complexity of agonistic and antagonistic muscle activities in both healthy control and LDH groups were quantified using sample entropy. As explained in Richman and Moorman [41], sample entropy is independent of the length of recorded signals, but it can display relative consistency under various circumstances. Taking this advantage, sample entropy has been applied on physiological time series analysis including diagnosis of Alzheimer’s disease [42]. Similarly, neuromuscular system can be considered as a complex dynamic system where sEMG of muscle activity on factors such as number of motor units of the muscles, muscle fiber conduction velocity, and discharge rate of action potential [43–45]. Algorithmically, sample entropy (S) of a recorded signal can be calculated as follows:

Given a time sequence data $\{ {\text{x}}({\text{i}})_{{\forall {\text{i}} = 1,2, \ldots ,{\text{K}}}} \}$, where ${\text{K }}$ is the total length of data, it is necessary to construct vectors of length m defined as:

8 $$\begin{aligned} {\text{X}}_{\text{i}} = \left[ {{\text{x}}_{\text{i}} , {\text{x}}_{{{\text{i}} + 1}} , \ldots \ldots , {\text{x}}_{{{\text{i}} + {\text{m}} - 1}} } \right] \hfill \\ \forall {\text{i}} = 1,2, \ldots ,{\text{K}} - {\text{m}} + 1 \hfill \\ \end{aligned}$$

Then, the probability that any of the vectors is similar with $\left( { X_{i} } \right)$ is calculated as given in Eq. 9:

9 $$Num_{i} \left( {m,r} \right) = \frac{{num\left( {d\left[ {X\left( i \right),X\left( j \right)} \right] \le r} \right)}}{K - m + 1}$$

where $num\left( {d\left[ {X\left( i \right),X\left( j \right)} \right] \le r} \right)$ is the number of data segments (*X*_*j*_) that are similar to other segments *X*_*i*_ with a constraint of:

10 $${\text{d}}\left[ {{\text{X}}\left( {\text{i}} \right),{\text{X}}\left( {\text{j}} \right)} \right] = \mathop {\max }\limits_{\begin{subarray}{l} h = 0 \sim m - 1 \\ \quad i \ne j \end{subarray} } \left| {x\left( {i + h} \right) - x(j + h)} \right| \le {\text{r}}$$

where ${\text{d}}\left( {X_{i} ,X_{j} } \right)$ is the maximal absolute difference between vectors *X*_*i*_ and *X*_*j*_ in their respective scalar components; r specifies the filter level (tolerance). If the distance between *X*_*i*_ and *X*_*j*_ is less than r, then the counter of vectors similar to *X*_*i*_ will increase by one. Then, the average probability is calculated as follows:

11 $$A^{m} \left( {\text{r}} \right) = \frac{1}{K - m + 1}\mathop \sum \limits_{i = 1}^{K - m + 1} Num_{i} (m,r)$$

The same process was repeated for the subseries of length m + 1 to calculate *B*^*m*+1^(r). As a final step, the sample entropy can be calculated:

12 $${\text{S}}\left( {{\text{m}},{\text{r}},{\text{K}}} \right) = - ln\frac{{B^{m + 1} (r)}}{{A^{m} (r)}}$$

High number of matches of length m and m + 1 increases the accuracy and confidence of sample entropy estimate [46]. Small m values and large r values result into an increased number of matches. However, as r increases, the probability of match will tend to 1, thus the quantified sample estimate will lose the discriminative ability. In the same vein, underlying physical process may be obscured as m decreases. Therefore, m values and r values should be rationally chosen for reliable estimate of sample entropy. To calculate sample entropy for each sEMG signal, values of the parameters m and r could be determined using one of the existing methods [47–49].

**Fig. 4:**
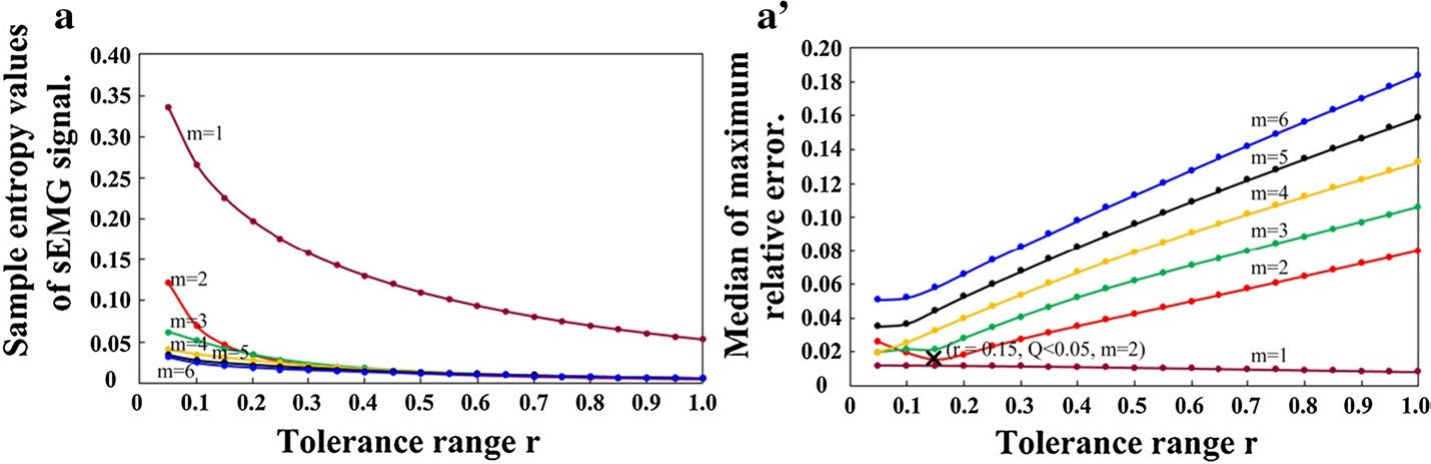
Optimal selections of parameters m and r. **a** Sample entropy is calculated over all-time series of sEMG signal, (**a′**) medial of maximum relative error that correspond to different m and r values are illustrated

To find rational m values, first sample entropy of each time-series was calculated for the combinations of m and r where m was in the range from 1 to 6 with a step of 1 and r ranged from 0.1 to 1 with a step of 0.05. Based on the values of m and r, the median sample entropy was calculated. The median sample entropy for sEMG signals is illustrated in Fig. 4a. It can be seen that the median sample entropy of all variables converges when m ≥ 2 for almost all r values.

To estimate the appropriate m and r values, conditional probability was calculated as:

13 $${\text{L}}\left( {{\text{m}},{\text{r}}} \right) = \frac{B(r)}{A(r)}$$

where ${\text{A}}({\text{r}})$ and ${\text{B}}\left( {\text{r}} \right)$ are the number of matches for subseries with length m and m + 1, respectively; while r is the tolerance value. The variance of L can be estimated as:

14 $$\partial_{L}^{2} = \frac{{L\left( {1 - L} \right)}}{B} + \frac{1}{{B^{2} }}\left[ {N_{A} - N_{B} \left( L \right)^{2} } \right]$$

where ${\text{N}}_{\text{A}}$ and ${\text{N}}_{\text{B}}$ are the number of pairs of matching templates of length m + 1 and m that are overlaps for a specified value of r, respectively. The tolerance value (r) can be determined by minimizing the expression of Eq. 15.

15 $${\text{Q}}\left( {{\text{m}},{\text{r}}} \right) = \hbox{max} \left( {\frac{{\partial_{L} \left( {m,r} \right)}}{{L\left( {m,r} \right)}}, \frac{{\partial_{L} \left( {m,r} \right)}}{{ - \log \left( {L\left( {m,r} \right)} \right)*L\left( {m,r} \right)}}} \right)$$

where ${\text{Q}}({\text{m}},{\text{r}})$ is the maximum relative error of sample entropy and L estimate.

The metric ${\text{Q}}\left( {{\text{m}},{\text{r}}} \right)$ can simultaneously penalize L close to 0 and 1. Hence, it becomes a tradeoff between accuracy and discriminative capability. Meanwhile, analysis from several experimental trials shows that setting the maximum relative error condition to a value lesser than 0.05 corresponds to a case where 95% confidence interval, which is 10% sample entropy estimation. The median between the maximum relative error for m ≥ 2 and all r values for sEMG signal were calculated. The medians obtained when the maximum error value was set as m = 2, 3, 4 are illustrated in Fig. 4a′. In order to obtain the best discriminative ability of sample entropy, the median of maximum relative error was set as 0.05, while the value for length (m) and tolerance (r) were set as 2 and 0.15, respectively.

### Statistical analysis

In this study, several statistical analysis were carried out to check if factors such as age, body weight, height, and BMI have influence on the muscle activities during the four movements. The statistical tests included one-sample *Kolmogorov*–*Smirnov normality test*. This analysis was carried out to verify whether each of the parameters: AEMG, CCR, sample entropy, determined from the acquired signals was normally distributed. Secondly, one-way analysis of variance (ANOVA) was used to determine whether there are any statistically significant differences (significant level p < 0.05) between the mean of age or BMI in both groups. Furthermore, independent sample *t test* was applied to examine the CCR measurements, agonist and antagonist muscle activities, and sample entropy of LDH against that of the healthy control group. Finally, *paired t test* was performed on the agonist and antagonist muscle activities to differentiate their contributions to the CCR during four types of movement. The statistical significant level was set to 0.05 in all the analysis.

## Results

The result from the statistical analysis shows that age and BMI do not have a significant influence on muscle activities of both LDH and healthy control group. Similarly, it was clear that the CCR, muscle activities (agonistic and antagonistic), and sample entropy values were not significantly different between genders. Asymptotic significances of the *Kolmogorov*–*Smirnov test* for CCR, agonist and antagonist activities, and sample entropy measurements (p > 0.05) indicated that all measurements complied with the normal distribution.

### Assessment of muscle coordination

**Fig. 5:**
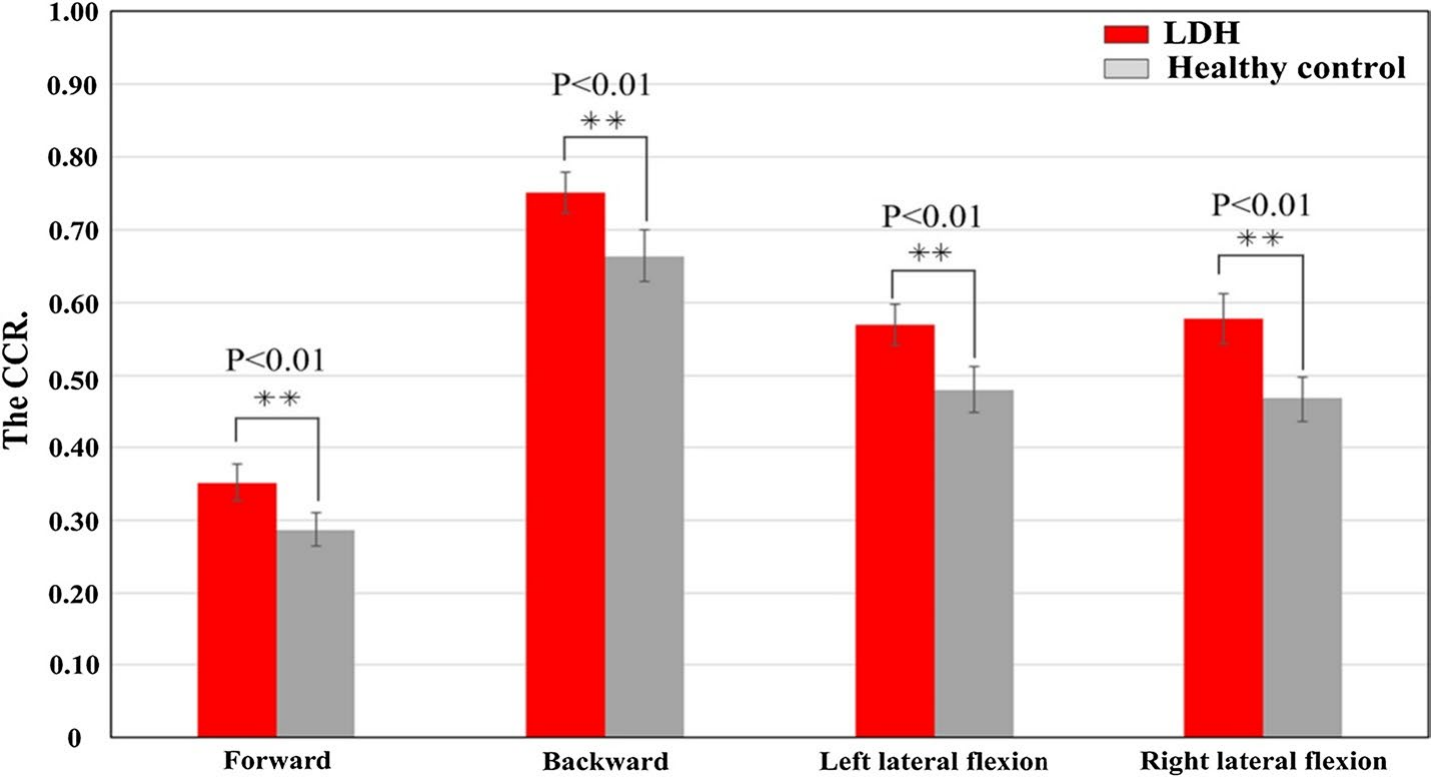
Magnitude of the CCR of both groups for each type of movement considered in this study. (*Significant difference (p < 0.05) between LDH and control groups. **Significant at 0.01 level.)

Analysis of CCR between the LDH and the healthy control group will not only reveal important features of neuromuscular control strategies, but it could also facilitate the design of appropriate training programs for the treatment of lumbar disorders. Comparing CCR values between the LDH and healthy subjects as shown in Fig. 5, the CCR of LDH obtained during the different types of movement was significantly higher than those of healthy controls. The specific values were: LDH (0.35 ± 0.02) vs. control (0.29 ± 0.02) in forward bending movement, LDH (0.75 ± 0.03) vs. control (0.66 ± 0.04) in backward bending actions, LDH (0.57 ± 0.03) vs. control (0.48 ± 0.03) in left lateral flexion actions, and LDH (0.58 ± 0.03) vs. control (0.47 ± 0.03) in right lateral flexion actions, all at p < 0.01.

Also, to analyze the influence of CCR in LDH subjects for each type of movement, we determined the change indicator (CI) which can be described as difference between the CCR of LDH group and that of the control group for a particular movement. For a given movement, the CI was computed as in Eq. 16.

16 $${\text{CI}} = \frac{{\left( {CCR_{LDH group} - CCR_{control group} } \right)}}{{CCR _{control group} }} \times 100\%$$

The CI values obtained for each of the four movements are presented in Table 3. The table shows that CCR in the right lateral flexion movement is higher than other three types of movement.

**Table 3:** The CI value during the four types of movement

	Forward bending	Backward bending	Left lateral flexion	Right lateral flexion
CI value (%)	22.58	13.11	18.55	23.79

### Agonistic and antagonistic muscles

**Fig. 6:**
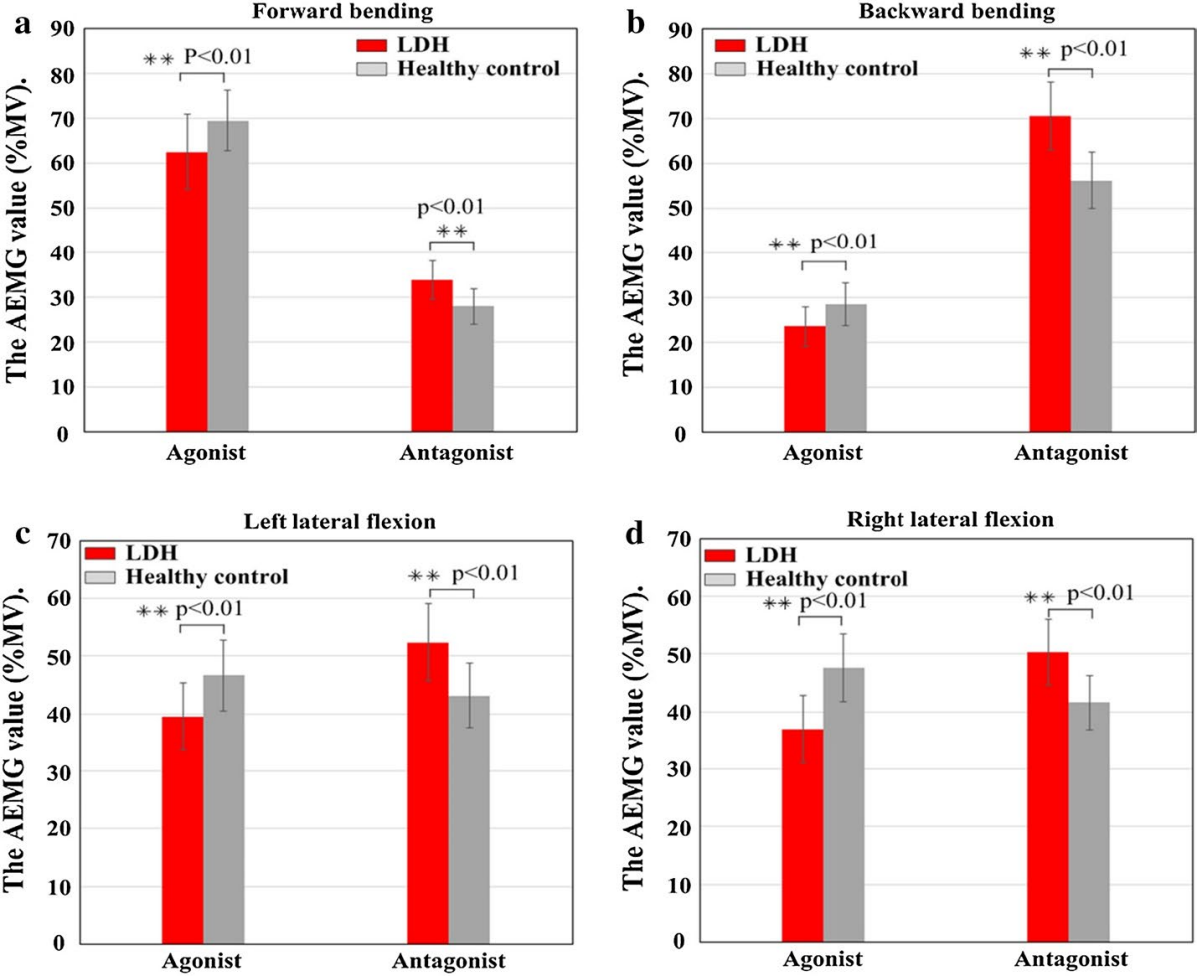
Analysis of agonistic and antagonistic muscle activities between LDH and healthy control during different types of movement. **a** Forward bending movements, **b** backward bending movements, **c** left lateral flexion actions and **d** right lateral flexion actions. (NB: heights of the agonist and antagonist are the sum of AEMG values for corresponding muscle activities during each type of movement. **Significant at 0.01 level.)

To analyze the effects of agonistic and antagonistic muscle activities on the CCR, independent *t test* was performed on the single-valued parameter (AEMG) obtained as mean amplitude of the sEMG signal from the LDH subjects and healthy controls during each of the four movements. As illustrated in Fig. 6, AEMG value of the agonistic muscle activities in LDH group was significantly lower than that of the controls during the four types of movement, while the antagonistic muscle activities in LDH group have a higher AEMG value compared to that of the control group. These data are presented in Table 4. Hence a significant difference (p < 0.01) was observed in agonistic muscle activities of subjects in both groups during the different types of movement (Fig. 6a–d, left), and antagonistic muscle activities between LDH patients and control group also had a significant difference (p < 0.01) for the same movement (Fig. 6a–d, right), respectively.

**Table 4:** AEMG of agonist and antagonist required for lumbar activities during the four movements

Movement	LDH (%MV)			Healthy control (%MV)		
	Agonist	Antagonist	Difference	Agonist	Antagonist	Difference
Forward bending	62.48 ± 8.32	33.78 ± 4.34	28.70**	69.45 ± 6.75	27.95 ± 3.90	41.50**
Backward bending	23.51 ± 4.45	70.67 ± 7.55	− 47.15**	28.46 ± 4.75	56.18 ± 6.30	− 27.72**
Left lateral flexion	39.59 ± 5.81	52.44 ± 6.75	− 12.84**	46.71 ± 6.16	43.20 ± 5.58	3.50*
Right lateral flexion	37.06 ± 5.88	50.37 ± 5.64	− 13.31**	47.64 ± 5.83	41.62 ± 4.68	6.02**

Values are mean ± standard deviation (SD), * significant level at 0.05; ** significant level at 0.01

Furthermore, to analyze the differences between agonist and antagonist muscle activities for LDH patients, and the differences between agonist and antagonist muscle activities for the control group, separately, *paired t test* of agonistic and antagonistic AEMG on CCR was observed for the four types of movement. As shown in Table 4, the AEMG value of antagonistic muscle activities was significantly greater than that of the agonistic muscles in LDH group during all types of movement, except for forward bending motion. However, in healthy controls, AEMG value of antagonistic muscle activities was significantly lower than that of agonistic muscles during forward bending, left lateral flexion and right lateral flexion actions.

### Sample entropy of sEMG signal in agonistic and antagonistic muscles

**Fig. 7:**
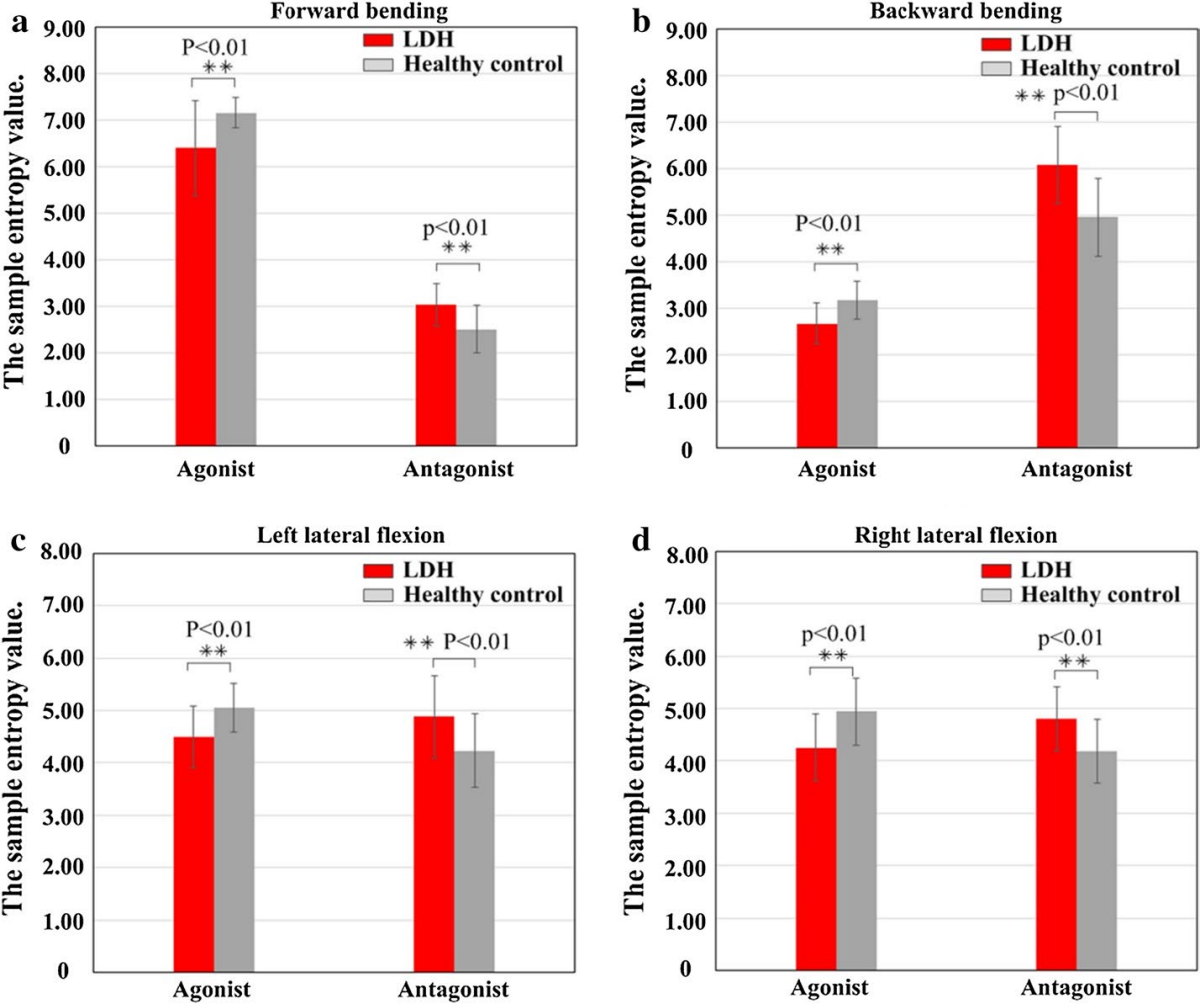
Analysis the sample entropy of the agonistic and antagonistic muscle activities between LDH and healthy control during four types of movement. **a** Forward bending movements, **b** backward bending movements, **c** left lateral flexion actions and **d** right lateral flexion actions. (The agonistic value stand for the sum of sample entropy value of all agonistic muscle sEMG at each movement pattern; the antagonistic value stands for the sum of sample entropy value of all antagonistic muscle sEMG at each movement pattern.)

The sample entropy was analyzed by running *independent t test* on sEMG signals obtained for agonistic and antagonistic muscle activities from both LDH and control groups during the four movements. Results of the analysis from each group are shown in Fig. 7.

A sample entropy value of 6.40 ± 1.02 was observed for agonistic muscle in the LDH group during the forward bending movement. Compared with that of the healthy controls, with a mean value of 7.15 ± 0.33, it is clear that the sample entropy value of the LDH group was significantly lower compared to healthy controls during the forward bending movement (p < 0.01). Similarly, in the backward bending movement, values of 2.68 ± 0.43 and 3.17 ± 0.40 were obtained for the LDH group and healthy control group, respectively. Correspondingly for left lateral flexion, sample entropy values of 4.49 ± 0.59 and 5.06 ± 0.47 were obtained for the LDH and control groups, respectively. Lastly, for right lateral flexion, the sample entropy values obtained for the LDH group and healthy controls were 4.25 ± 0.64 and 4.95 ± 0.64, respectively. Typically, these are as shown in Fig. 7.

Similarly, sample entropy value of the antagonistic muscle activities in LDH group was significantly greater than that of the controls (p < 0.01). In the case of forward bending movement, mean values of 3.04 ± 0.45 and 2.51 ± 0.50 were achieved for the LDH and control groups, respectively. For backward bending movement, the mean values observed for the LDH and control groups were 6.08 ± 0.83 and 4.96 ± 0.84, respectively. However, during left lateral flexion, mean values of 4.88 ± 0.79 and 4.23 ± 0.70 were achieved for the LDH and control groups, respectively; while both group possessed 4.80 ± 0.61 and 4.19 ± 0.61, respectively, during right lateral flexion movements. Hence a significant difference was observed in agonistic and antagonistic muscle activities of subjects in both groups during the different types of movement when compared with the control group with p < 0.01 for all movements.

## Discussion

In this study, we carried out experiments to explore the co-contraction of trunk muscles between LDH and healthy control groups in different types of movement which includes forward bending, backward bending, left lateral flexion, and right lateral flexion. Sample entropy was applied to predict stereotypical recruitment changes in the lumbar muscles during the movements. Results obtained from the experimental study confirmed that the co-contraction ratio of LDH subjects was significantly higher for each of the movements as compared to that of the healthy controls. Also, statistical analysis of sEMG signals acquired from both groups reflects that the agonistic and antagonistic muscle activities and muscle recruitment pattern in both groups were significantly different.

Furthermore, it was observed that the co-contraction ratio of LDH subjects is higher than that of the healthy controls for the four types of movement. This could be attributed to higher spine loads which is commonly found in patients with herniated disks. As a result, LDH patients are more susceptible to lumber damage when performing more delicate movements. In addition, change indicator was used to describe the difference between the two groups, and it was observed that the CI value is greater than 13% in subjects with LDH compared to controls for all movements. In fact, the percent change in co-contraction ratio from the LDH to control group was 23.79% during right lateral flexion movement. This value is higher than what was observed for the remaining three movements, as presented in Table 3. Thus, such change in co-contraction ratio may indicate a risk factor for the LDH subjects. Increased antagonistic muscle activity found in the LDH group for the four types of movement may be as a result of higher imposed trunk moments that accompany the motions. Furthermore, the theoretical analysis predicts that stabilizing agonistic muscle activity should decline with increased trunk moment [50]. However, when trunk moments increase, the muscles have to increase their output to offset the moment, resulting in higher lumbar spine load. Therefore, the high co-contraction in the LDH group could be as a result of high spine loads or vice versa. Thus, as part of rehabilitation program for LDH patients, exercises with reduced lumbar spine load should be selected for effective rehabilitation keep-fit training. This will eliminate or minimize the risk of recurrence lumbar spine problems. Hence, it is paramount to advise patients not to perform complicated programs which could increase their lumbar spine loads.

Moreover, as suggested for numerous motor control changes [51], muscle co-contraction depends on spine control demands to maintenance of lumbar spine stabilization [52]. The present study provides clear evidence that co-contraction ratio in LDH subjects is higher for the four movements compared to that of the healthy controls, and that agonistic and antagonistic activities are controlled differently. Specifically, the co-contraction ratio of participants with LDH increased in the four movements since the agonistic activity reduced with noticeable increase in the antagonistic muscle activities. Hence, this indicates that among the four movements, the agonistic muscles were not sufficiently activated due to control demands. In alignance with hitherto, profound activation of the antagonistic muscles during the four movements shows a quite imbalance in trunk muscles of LDH subjects. In healthy persons, the muscles around the lumbar spine serve to stabilize the spine and contribute to maintain the posture. Similarly, the agonistic and antagonistic muscle activities around the spine also help in coordinating the movements required for stability of the spine [53, 54]. The co-contraction ratio may be increased because of reduced passive spine stability, distorted proprioceptive input, or reduced lumbar muscle force with pains from spinal injury [55]. Thus, insufficient activation of agonistic muscles and over-activation of antagonistic muscles imply that neuromuscular control in the LDH subjects could provide relatively less protection for the patients. It is important to state that difference in the muscle activities observed from results of our experimental study could indicate multifaceted variations in motor control of the LDH subjects. Hence, this reflects different propensity for healthy control group to co-contract flexor and extensor lumbar muscles during each of the movements. Furthermore, as presented in Table 4, antagonistic muscles play major roles in the co-contraction needed for lumbar activities in LDH subjects during the movements, except for the forward bending motion. Conversely, agonistic muscles contribute vitally to the co-contraction needed for lumbar activities in healthy control group during other movements except backward bending motion. Abnormal activations of trunk muscles or neuromuscular control errors seems to affect the spine’s stability. Therefore, this might be a reason why disc herniation recurs in patients with confirmed LDH.

Trends in co-contraction ratio of patients with LDH, including the agonistic and antagonistic muscle data presented in the current study may be explained by recruitment patterns necessary to maintain lumbar spine stability [56]. By calculating sample entropy values of sEMG in agonistic and antagonistic muscles, LDH subjects used for this study exhibited greater antagonistic muscle activities and lower agonistic muscle activities compared with the controls during the four movements. A reason for the greater sample entropy of antagonistic muscles and lower sample entropy of agonistic muscles in patients with LDH can be explained with the differences in the muscle recruitment of those subjects compared to the healthy controls. Since different motor units of the muscle may be recruited in a specific fashion during locomotion [57], higher antagonistic muscle activities tend to be more complex with oscillatory modes in the original signals. This yields greater sample entropy values. On the contrary, lower agonistic activities tend to become more regular with smooth oscillations. Consequently, this results in smaller sample entropy values. On this basis, the reduced sample entropy of agonistic muscles and the increased sample entropy of antagonistic muscles were found in patients with LDH compared to control group, respectively. Overall, the muscle recruitment pattern was significantly different as revealed in results of the statistical analysis.

As discussed above, evaluation of co-contraction ratio, AEMG, and sample entropy in the LDH and healthy control groups appeared to aid the identification of risk factors for LDH patients. Hence, inadequate agonist/antagonist coordination and abnormal oscillatory modes would seem to clarify some mechanical causes of lumbar spinal diseases or has the potential to improve the current treatment options. Co-contraction of lumbar spine increased when agonistic decreased and antagonistic increased in patients with LDH. So, during rehabilitation, LDH patients should appear to strengthen agonistic muscle activities and weaken antagonistic muscle activities by choosing the appropriate rehabilitation training. In the future, further research will be carried out to provide more insights into those mechanisms for the purpose of developing better rehabilitative programs.

Our study has several limitations. We only recruited 26 subjects with slight LDH who can perform the four movements voluntarily. Patients with complicated LDH were not considered so as to avoid worries about possibility of damaging their lumbar spine when performing the movements. Second, all subjects were implored to ensure they achieve a consistent amplitude of movement during the four movements; however, there were still a few variations in the amplitude attained by each subject. In the future, we will investigate the influence of these variations on the results. Similarly, adaptive experiments based on different LDH levels will be carried out with emphasis on revealing the recurrent factors of LDH or relationship between disk pathology, symptoms, and disability. Furthermore, dependent measures such as the co-contraction ratio, and AEMG will be examined for the ability to screen for LDH.

## Conclusions

The main aim of this study is to explore the difference of trunk muscle co-contraction between patients with LDH and healthy control groups during four types of movement namely: forward bending, backward bending, left lateral flexion and right lateral flexion movements. For each movement made by each subject, average electromyogram (mean of amplitudes of sEMG signals from several trials) was calculated, and used to quantify the co-contraction ratio of the movements. Sample entropy was applied to predict oscillatory model changes in antagonistic and agonistic muscle recruitment during the four movements. From the results of this study, it can be concluded that the co-contraction ratio of trunk muscles in LDH patients was significantly higher than that of healthy controls during the four movements with a statistically significant difference (p < 0.05). In the same vein, this study showed that agonistic muscle activities of the LDH patients were lower when compared to those of the healthy controls, while the antagonistic muscle activities were higher for LDH patients during the four movement. Furthermore, the sample entropy value of agonistic muscles in LDH patients exhibited lower level than that of the healthy controls, while that of the antagonistic muscles were greater in LDH patients during the four movements. The right lateral flexion movements showed a greater potential risk of lumbar spine instability due to the cost of load.

## Abbreviations

LDH: lumbar disc herniation
LM: lumbar multifidus
CT: computed tomography
ANOVA: analysis of variance
ECG: electrocardiographic
AEMG: average electromyography
EMG: electromyography
IO/TrA: internal oblique/transversus abdomini
MRI: magnetic resonance imaging
VAS: visual analogue scale
MVC: maximum voluntary contraction
CCR: co-contraction ratio
EO: external oblique
sEMG: surface electromyography
BMI: body mass index
WPES: wavelet packet energy spectrum
MV: maximum value
CI: change indicator

## References

[1] Watanabe S, Kobara K, Yoshimura Y, Osaka H, Ishida H (2014). Influence of trunk muscle co-contraction on spinal curvature during sitting. J Back Musculoskelet Rehabil.

[2] Watanbe S, Kobara K, Ishida H, Eguchi A (2010). Influence of trunk muscle co-contraction on spinal curvature during sitting cross-legged. Electromyogr Clin Neurophysiol.

[3] Watanabe S, Eguchi A, Kobara K, Ishida H (2007). Influence of trunk muscle co-contraction on spinal curvature during sitting for desk work. Electromyogr Clin Neurophysiol.

[4] Panjabi MM (1992). The stabilizing system of the spine. Part II. Neutral zone and stability hypothesis. J Spinal Disord.

[5] Arjmand N, Shirazi-Ad A, Parnianpour M (2008). Relative efficiency of abdominal muscles in spine stability. Comput Methods Biomech Biomed Eng.

[6] Graham RB, Oikawa LY, Ross GB (2014). Comparing the local dynamic stability of trunk movements between varsity athletes with and without non-specific low back pain. J Biomech.

[7] Baek YJ, Jung YJ, Son JL, Lim OB, Yi CH (2017). Comparison of muscle activity and trunk compensation during modified push-up plus exercises in individuals with scapular winging. Isokinet Exerc Sci.

[8] Bergmark A (1989). Stability of the lumbar spine: a study in mechanical engineering. J Acta Orthop Scand.

[9] Noormohammadpour P, Khezri AH, Linek P, Mansournia MA, Hassannejad A, Younesian A, Farahbakhsh F, Kordi R (2016). Comparion of lateral abdominal muscle thickness and cross sectional area of multifidus in adolescent soccer players with and without low back pain: a case control study. Asian J Sports Med.

[10] Bogduk N (2015). Clinical anatomy of the lumbar spine and sacrum.

[11] Danneels L, Vleeming A, Mooney V, Stoeckart R (2007). Clinical anatomy of the lumbar multifidus. Movement, stability and lumbopelvic pain: integration of research and therapy.

[12] Seo JY, Roh YH, Kim YH, Ha KY (2016). Three-dimensional analysis of volumetric changes in herniated discs of the lumbar spine: does spontaneous resorption of herniated discs always occur?. Eur Spine J.

[13] Haro H (2014). Translational research of herniated discs: current status of diagnosis and treatment. J Orthop Sci.

[14] Grunert P, Reyes PM, Newcomb AGUS, Towne SB, Kelly BP, Theodore N, Hart R (2016). Biomechanical evaluation of lumbar decompression adjacent to instrumented segments. Neurosurgery.

[15] Ekstrom RA, Donatelli R, Carp KC (2008). Electromygraphic analysis of core trunk, hip, and thigh muscles during 9 rehabilitation exercises. J Orthop Sports Phys Ther.

[16] Truszczynska A, Dobrzynska M, Trzaskoma Z, Drzalgrabiec J, Tarnowski A (2016). Assessment of postural stability in patients with lumbar spine chronic disc disease. Acta Bioeng Biomech.

[17] Kim JY, Ryu DS, Paik HK, Ahn SS, Kang MS, Kim KH, Park JY, Chin DK, Kim KS, Cho YE, Kuh SU (2016). Paraspinal muscle, facet joint, and disc problems: risk factors for adjacent segment degeneration after lumbar fusion. Spine J.

[18] Cheng CH, Lin KH, Wang JL (2008). Co-contraction of cervical muscles during sagittal and coronal neck motions at different movement speeds. Eur J Appl Physiol.

[19] Fallah-Yakhdani HR, Abbasi-Bafghi H, Meijer OG, Bruijn SM, van den Dikkenberg N, Benedetti MG, van Dieen JH (2012). Determinants of co-contraction during walking before and after arthroplasty for knee osteoarthritis. Clin Biomech.

[20] Hodges PW, van den Hoorn W, Wrigle TV, Hinman RS, Bowles KA, Cicuttini F, Wang YY, Bennell K (2016). Increased duration of co-contraction of medical knee muscles is associated with greater progression of knee osteoarthritis. Man Ther.

[21] Piscitelli D, Falaki A, Solnik S, Latash ML (2017). Anticipatory postural adjustments and anticipatory synergy adjustments: preparing to a postural perturbation with predictable and unpredictable direction. Exp Brain Res.

[22] Blache Y, Maso FD, Desmoulins L, Plamondon A, Begon M (2015). Superficial shoulder muscle co-activations during lifting tasks: influence of lifting height, weight and phase. J Electromyogr Kinesiol.

[23] Neves Rosa MC, Marques A, Demain S, Metcalf CD (2014). Lower limb co-contraction during walking in subjects with stroke: a systematic review. J Electromyogr Kinesiol.

[24] Lustosa LP, Ocarino JM, Percope de Andrade MA, de Melo Pertence AE, Bittencourt NFN, Fonseca ST (2011). Muscle co-contraction after anterior cruciate ligament reconstruction: influence of functional level. J Electromyogr Kinesiol.

[25] Granata KP, Lee PE, Franklin TC (2005). Co-contraction recruitment and spinal load during isometric trunk flexion and extension. Clin Biomech.

[26] Silvestri G, Schinkel-lvy A, Drake JDM (2013). Asseeement of trunk muscle co-contraction during typical occupational movement tasks. Occup Ergon.

[27] Schinkel-lvy A, Nairn BC, Drake JDM (2013). Investigation of trunk muscle co-contraction and its association with low back pain development during prolonged sitting. J Electromyogr Kinesiol.

[28] Konrad P. The ABC of EMG a practical introduction to kinesiological electromyography. Scottsdale: Noraxon Inc. Version 1.0; 2005.

[29] Ronager J, Christensen H, Fuglsang-Frederiksen A (1989). Power spectrum analysis of the EMG pattern in normal and diseased muscles. J Neurol Sci.

[30] Zhang YY, Wang G, Teng CL, Sun ZJ, Wang J (2014). The analysis of hand movement distinction based on relative frequency band energy method. Biomed Res Int.

[31] Kuiken TA (2004). The use of targeted muscle reinnervation for improved myoelectric prosthesis control in a bilateral shoulder disarticulation amputee. Prosthet Orthot Int.

[32] Sahambi J, Tandon S, Bhatt R (1997). Using wavelet transforms for ECG characterization. An on-line digital signal processing system. IEEE Eng Med Biol Mag.

[33] Thakor NV, Webster JG, Tompkins WJ (1984). Estimation of QRS complex power spectra for design of a QRS filter. IEEE Trans Biomed Eng.

[34] Behrens M, Mau-Moeller A, Heise S, Skripitz R, Bader R, Bruhn S (2015). Alteration in neuromuscular function of the plantar flexors following caffeine ingestion. Scand J Med Sci Sports.

[35] Halaki M, Ginn K. Normalization of EMG signals: to normalize or not to normalize and what to normalize to? Rijeka: Intech. 2012; chapter 7. p. 176–94.

[36] Cheng CH, Cheng HYK, Chen CPC, Lin KH, Liu WY, Wang SF, Hsu WL, Chuang YF (2014). Altered co-contraction of cervical muscles in young adults with chronic neck pain during voluntary neck motions. J Phys Ther Sci.

[37] Falconer K, Winter DA (1985). Quantitative assessment of co-contraction at the ankle joint in walking. Electromyogr Clin Neurophysiol.

[38] Choi H (2003). Quantitative assessment of co-contraction in cervical musculature. Med Eng Phys.

[39] Shin G, D’Souza C (2010). EMG activity of low back extensor muscles during cyclic flexion/extension. J Electromyogr Kinesiol.

[40] Farahpour N, Younesian H, Bahrpeyma F (2015). Electromyographic activity of erector spinae and external oblique muscles during trunk lateral bending and axial rotation in patients with adolescent idiopathic scoliosis and healthy subjects. Clin Biomech.

[41] Richman JS, Moorman JR (2000). Physiological time-series analysis using approximate entropy and sample entropy. Am J Physiol Heart Circ Physiol.

[42] Cao YZ, Cai LH, Wang J, Yu HT, Cao YB, Liu J (2015). Characterization of complexity in the electroencephalograph activity of Alzheimer’s disease based on fuzzy entropy. Chaos.

[43] Roeleveld K, Stegeman DF, Vingerhoets HM, Vanoosterom A (1997). Motor unit potential contribution to surface electromyography. Acta Physiol Scand.

[44] Farina D, Merletti R (2004). Estimation of average muscle fiber conduction velocity from two-dimensional surface EMG recordings. J Neurosci Methods.

[45] Farina D, Falla D (2009). Discharge rate of sternohyoid motor units activated with surface EMG feedback. J Neurophysiol.

[46] Cui D, Wang JH, Bian ZJ, Li QL, Wang L, Li XL (2015). Analysis of entropies based on empirical mode decomposition in amnesic mild cognitive impairment of diabetes mellitus. J Innov Opt Health Sci.

[47] Xie HB, He WX, Liu H (2008). Measuring time series regularity using nonlinear similarity-based sample entropy. Phys Lett A.

[48] Zhang X, Zhou P (2012). Sample entropy analysis of surface EMG for improved muscle activity onset detection against spurious background spikes. J Electromyogr Kinesiol.

[49] Sharma R, Pachori RB, Acharya UR (2015). Application of entropy measures on intrinsic mode functions for the automated identification of focal electroencephalogram signals. Entropy.

[50] Cholewicki J, Panjabi MM, Khachatryan A (1997). Stabilizing function of trunk flexor-extensor muscles around a neutral spine posture. Spine.

[51] Masse-Alarie H, Neaulieu LD, Preuss R, Schneider C (2017). Repetitive peripheral magnetic neurostimulation of multifidus muscles combined with motor training influence spine motor control and chronic low back pain. Clin Neurophysiol.

[52] Dhooge R, Hodges P, Tsao H, Hall L, MacDonald D, Danneels L (2013). Altered trunk muscle coordination during rapid trunk flexion in people in remission of recurrent low back pain. J Electromyogr Kinesiol.

[53] Lee N, Kang H, Shin G (2015). Use of antagonist muscle EMG in the assessment of neuromuscular health of the low back. J Physiol Anthropol.

[54] Reeves NP, Popovich MJ, Priess MC, Cholewicki J, Choi J, Radcliffe CJ (2014). Reliability of assessing trunk motor control using position and force tracking and stabilization tasks. J Biomech.

[55] Jacobs JV, Lomond KV, Hitt JR, DeSarno MJ, Bunn JY, Henry SM (2016). Effects of low back pain and of stabilization or movement-sustem-impairment treatments on induced postural responses: a planned secondary analysis of a randomised controlled trial. Man Ther.

[56] Costa M, Goldberger AL, Peng CK (2002). Multiscale entropy analysis of complex physiologic time series. Phys Rev Lett.

[57] Zijdewind I, Bakels R, Thomas CK (2014). Motor unit firing rates during spasms in thenar muscles of spinal cord injured subjects. Front Hum Neurosci.

